# Outcome of the Ponseti Method for Treatment of Idiopathic Congenital Clubfoot

**DOI:** 10.7759/cureus.79387

**Published:** 2025-02-20

**Authors:** Raja Muhammad Mussab, Syed Muhammad Zohaib Raza, Ramsha Kirmani, Kazim Hussain, Shehanshah Muhammed Arqam

**Affiliations:** 1 Orthopaedics and Trauma, Jinnah Postgraduate Medical Centre, Karachi, PAK; 2 Orthopaedics and Trauma, Russell Hall Hospital, Dudley, GBR; 3 Orthopaedics, The Indus Hospital, Karachi, PAK

**Keywords:** conservative and surgical treatment, idiopathic congenital clubfoot, pirani score, ponseti casting, ponseti's method

## Abstract

Introduction

Idiopathic congenital clubfoot is a complex deformity that requires treatment to reduce the deformity to a flexible, plantigrade, and painless foot. The Ponseti method has gained international prominence due to its effectiveness and reduction in the need for surgical procedures. The aim of this study is to determine the outcome of the Ponseti method in patients with idiopathic congenital clubfoot, which will generate local evidence.

Methods

A descriptive cross‐sectional study was conducted in the Department of Orthopaedics at Jinnah Postgraduate Medical Centre (JPMC) Karachi over a six‐month period, from September 6, 2022, to March 7, 2023. Participants were included if they were children under three years of age, presented with idiopathic congenital clubfoot, and had a Pirani score of 2 or higher. The outcome was assessed immediately after the completion of treatment and after four months by using the Pirani score. Results were graded as excellent (Pirani score <1), good (score 1-2), and poor (score >2). Baseline demographic characteristics and Pirani score were recorded immediately after completion of treatment and after four months. Effect modifiers such as age, gender, weight, residence, and baseline Pirani score were controlled through stratification. Post-stratification Chi-square test was applied taking p-value ≤0.05 as significant.

Results

Of 91 neonates, the mean age of the neonates was 1.44 ±0.66 years. There were 51 (56%) males and 40 (44%) females. The Pirani score immediately after the treatment was 1.61 ± 1.38. Excellent outcome immediately after the treatment was observed in 43 (47.3%), good in 26 (28.6%), and fair in 22 (24.2%). At four months follow-up, the Pirani score was 0.91 ± 0.81. Excellent outcome immediately after the treatment was observed in 63 (69.2%), good in 23 (25.3%), and fair in five (5.5%).

Conclusion

The Ponseti method is an effective and reliable treatment for idiopathic congenital clubfoot at a tertiary care hospital. The intervention achieves marked improvement in deformity correction and also sustains clinical benefits over the short-term follow-up period. Future research with extended follow-up periods and broader sample sizes is needed to further evaluate long-term functional outcomes.

## Introduction

Congenital clubfoot is a unilateral or bilateral malformation associated with abnormalities in type III collagen production. These abnormalities can be due to genetic mutations affecting collagen synthesis and structure. It can be classified as postural, idiopathic, and teratogenic [1,2]. Musculoskeletal changes are the main features of this pathology, as they impair the stages of normal child development. This congenital deformity is characterized by a rigid foot in the varus and equinus positions of the hindfoot (calcaneus), cavism, and supination of the midfoot, associated with adduction of the forefoot [1-3]. Congenital clubfoot can occur in isolation, being idiopathic, or associated with other congenital deformities, such as neuromuscular diseases (arthrogryposis) and recently also associated with the Zika virus syndrome [4,5].

Clubfoot affects approximately one to two out of every 1,000 live births and globally is three times more prevalent in males [6]. Various factors including genetic predisposition, gestational abnormalities, and a range of histological changes are thought to contribute to its development, though the exact aetiology remains elusive [7].

Congenital idiopathic clubfoot is a complex deformity that presents significant challenges in correction [3]. The goal of treatment is to reduce the deformity to a flexible, plantigrade, and painless foot, which facilitates the child's development [8]. Initially, conservative methods are used to solve the problem. For this purpose, two main methods were established, namely the Kite method and the Ponseti method [9]. Most orthopaedic surgeons have increasingly adopted the Ponseti method as the gold standard [10].

The Ponseti method gained international prominence due to its effectiveness and reduction in the need for surgical procedures. When comparing the methods, the Ponseti method is superior in improving the prognosis and maintenance of therapeutic changes in the long term [11,12]. The Ponseti method consists of placing a plaster cast with weekly changes with correct manipulation so that there is an effective and sequential reduction of deformities [10]. It allows weekly assessment using the Pirani scale, used in children diagnosed with clubfoot up to two years of age who have not undergone surgery and is graded from 0 to 1, normal to severely abnormal in each of six assessment indexes [12]. The duration of treatment varies, but there is an assessment up to four years of age, justified by the end of the growth of type III collagen and consequently a pause in the genetic alteration that affected it.

In relation to the effectiveness of the Ponseti method, previously various studies have been done at the international level, but we found differences in the results of the literature [11]. This variation may be due to differences in demographic characteristics and criteria used for assessment of outcomes [11]. Furthermore, local studies on this topic are very limited. Therefore, the aim of this study is to determine the outcome of the Ponseti method in patients with idiopathic congenital clubfoot, which will generate local evidence. Moreover, the results of this study will help the surgeon choose appropriate evidence-based techniques in order to reduce later complications.

## Materials and methods

A descriptive cross‐sectional study was conducted in the Department of Orthopaedics at Jinnah Postgraduate Medical Centre (JPMC) Karachi over a six‐month period, from September 6, 2022, to March 7, 2023. The study was conducted after obtaining approval from the College of Physicians and Surgeons of Pakistan (approval CPSP/REU/OSG-2021-186-2551).

Participants were included if they were children under three years of age, regardless of gender, who presented with idiopathic congenital clubfoot and had a Pirani score of 2 or higher at the time of presentation, and who were undergoing treatment with the Ponseti method. Conversely, children presenting with secondary clubfoot, those with previously operated feet, feet with local wounds, or patients who were lost to follow-up were excluded from the study.

The sample size was determined using the WHO sample size calculator [13], based on a 75% frequency of excellent outcomes observed in patients treated with the Ponseti method, a margin of error of 7%, and a confidence level of 95%, yielding a required sample size of 147 participants. A non-probability consecutive sampling technique was employed to enroll patients throughout the study period. Consecutive sampling is time-effective and easy to implement but it lacks random selection, which can potentially introduce selection bias [14].

All children presenting with idiopathic congenital clubfoot and fulfilling the inclusion criteria were enrolled in the study. Prior to enrolment, written informed consent was taken from parents/guardians of the children. Detailed demographic and baseline details were taken at the time of enrolment. Before starting treatment, the baseline Pirani score was noted in a predesigned questionnaire. Treatment commenced as soon as the skin condition allowed and involved gentle manipulation of the foot, followed by the sequential application of long-leg plaster casts on a weekly basis, in accordance with Ponseti's protocol and without the use of anaesthesia. In every case, the initial focus was on correcting the cavus by supinating the forefoot and dorsiflexing the first metatarsal. To address the varus and adduction components, the foot, maintained in supination, was subsequently abducted, with counter-pressure applied against the talar head using the thumb. Four long leg casts were changed weekly after proper manipulation of the foot was applied. The outcome was assessed immediately after the completion of treatment and after four months by using the Pirani score. The Pirani scoring system, which evaluates six parameters each assigned a value of 0, 0.5, or 1, yields a total score ranging from 0 to 6. Based on this, outcomes were classified as excellent (score below 1), good (score between 1 and 2), or poor (score above 2).

Data was entered and analysed using IBM SPSS Statistics for Windows, version 24.0 (IBM Corp., Armonk, NY, USA). Mean ± SD was reported for quantitative variables such as age, baseline Pirani score, and weight, whereas qualitative variables such as gender, residence, and outcome immediately after completion of treatment and after four months were presented as frequency and percentage. Effect modifiers such as age, gender, weight, residence, and baseline Pirani score were controlled through stratification. Post-stratification Chi-square test was applied taking p-value ≤0.05 as significant.

## Results

A total of 91 neonates were enrolled in this study, with demographic data summarised in Table 1. The mean age of the participants was 1.44 ± 0.66 years, with a slight male predominance (56.04% male vs. 43.96% female). The average weight recorded was 12.14 ± 1.56 kg. Additionally, the majority of the neonates resided in urban areas (63.74%), while 36.26% came from rural settings.

**Table 1:** Demographic characteristics of the participants (n=91)

Characteristics	Result (Mean ± SD or n (%))
Age (years)	1.44 ±0.66
Gender	
Male	51 (56.04%)
Female	40 (43.96%)
Weight (kg)	12.14 ±1.56
Residence Status	
Rural	33 (36.26%)
Urban	58 (63.74%)

Table 2 outlines the treatment parameters and outcomes following the application of the Ponseti method. At baseline, the mean Pirani score was 3.77 ± 0.73, which reflects the severity of the initial deformity. On average, 3.84 ± 1.01 casts were applied per patient. Immediately post-treatment, the mean Pirani score decreased significantly to 1.61 ± 1.38, with outcomes distributed as fair in 22 cases (24.16%), good in 26 cases (28.57%), and excellent in 43 cases (47.25%). Notably, at the four-month follow-up, the improvement was sustained and further enhanced: the mean Pirani score dropped to 0.91 ± 0.81, and the proportion of excellent outcomes increased to 69.23% (63 cases), with only 5.50% (five cases) classified as fair and 25.27% (23 cases) as good. These findings underscore the effectiveness of the Ponseti method in achieving significant clinical improvement in neonates with idiopathic congenital clubfoot over the short-term follow-up period.

**Table 2:** Treatment parameters and outcomes following the Ponseti method (n=91)

Parameter/Outcome	Result (Mean ± SD or n (%))
Pirani score at baseline	3.77 ± 0.73
Number of casts applied	3.84 ± 1.01
Pirani score immediately after treatment	1.61 ± 1.38
Outcome immediately after treatment	
Fair	22 (24.16%)
Good	26 (28.57%)
Excellent	43 (47.25%)
Pirani score after 4 months	0.91 ± 0.81
Outcome after 4 months	
Fair	5 (5.50%)
Good	23 (25.27%)
Excellent	63 (69.23%)

Table 3 presents a comparative analysis of treatment outcomes immediately after the Ponseti method and at four months post-treatment, stratified by various patient characteristics. In terms of age, while the immediate outcomes between children aged two or under and those between two and three years did not differ significantly (p=0.110), a marked improvement was noted at four months, with the older group exhibiting a significantly higher proportion of excellent outcomes (88.0% vs. 62.1%; p<0.001).

**Table 3:** Comparison of outcome immediately after treatment and after four months (n=91) p-value ≤0.05 is significant.

Variable	Category	Outcome Immediately After Treatment			p-value	Outcome After 4 Months			p-value
		Excellent	Good	Fair		Excellent	Good	Fair	
Age (years)	≤2	27 (40.9%)	20 (30.3%)	19 (28.8%)	0.110	41 (62.1%)	23 (34.8%)	2 (3.0%)	<0.001
	>2 to <3	16 (64.0%)	6 (24.0%)	3 (12.0%)		22 (88.0%)	0 (0%)	3 (12.0%)	
Gender	Male	17 (42.5%)	13 (32.5%)	10 (25.0%)	0.688	28 (70.0%)	10 (25.0%)	2 (5.0%)	0.980
	Female	26 (51.0%)	13 (25.5%)	12 (23.5%)		35 (68.6%)	13 (25.5%)	3 (5.9%)	
Weight (kg)	≤12	28 (48.3%)	16 (27.6%)	14 (24.1%)	0.956	40 (69.0%)	17 (29.3%)	1 (1.7%)	0.075
	>12	15 (45.5%)	10 (30.3%)	8 (24.2%)		23 (69.7%)	6 (18.2%)	4 (12.1%)	
Residence	Urban	11 (33.3%)	14 (42.4%)	8 (24.2%)	0.061	21 (63.6%)	11 (33.3%)	1 (3.0%)	0.344
	Rural	32 (55.2%)	12 (20.7%)	14 (24.1%)		42 (72.4%)	12 (20.7%)	4 (6.9%)	
Baseline Pirani Score	≤4	38 (50.0%)	24 (31.6%)	14 (18.4%)	0.014	56 (73.7%)	17 (22.4%)	3 (3.9%)	0.088
	>4	5 (33.3%)	2 (13.3%)	8 (53.3%)		7 (46.7%)	6 (40.0%)	2 (13.3%)	
Number of Casts Applied	≤4	43 (61.4%)	23 (32.9%)	4 (5.7%)	<0.001	62 (88.6%)	7 (10.0%)	1 (1.4%)	<0.001
	>4	0 (0%)	3 (14.3%)	18 (85.7%)		1 (4.8%)	16 (76.2%)	4 (19.0%)	

Gender did not appear to influence the outcomes, as both male and female groups showed comparable results immediately post-treatment (p=0.688) and at the four-month follow-up (p=0.980). Similarly, when stratified by weight (≤12 kg vs. >12 kg), no significant differences were observed in immediate outcomes (p=0.956) and a marginal difference at four months (p=0.075), suggesting weight had minimal impact on treatment efficacy.

The analysis based on residence status indicated that although urban and rural patients had borderline differences immediately after treatment (p=0.061), these differences did not reach statistical significance at four months (p=0.344). Notably, baseline severity, as measured by the Pirani score, played a crucial role; patients with a lower baseline score (≤4) achieved significantly better immediate outcomes compared to those with a higher baseline score (>4) (p=0.014), although this distinction was not statistically significant at the four-month mark (p=0.088).

Perhaps the most striking finding was related to the number of casts applied. Patients requiring four or fewer casts demonstrated significantly superior outcomes both immediately after treatment and at the four-month follow-up (p<0.001 for both time points) compared to those who needed more than four casts. These findings underscore the importance of early intervention and effective casting in achieving optimal outcomes with the Ponseti method, as detailed in Table 3.

Overall, the Ponseti method yielded substantial improvements in the clinical outcomes of neonates with idiopathic congenital clubfoot, as demonstrated by a significant reduction in the Pirani score both immediately following treatment and at the four‐month follow-up (Table 2). Notably, a marked increase in the proportion of excellent outcomes was observed over time, underscoring the sustained efficacy of the method. Stratified analysis (Table 3) revealed that while demographic factors such as gender, weight, and residence had minimal impact on treatment success, key clinical parameters - including baseline severity and the number of casts applied - played a pivotal role. Specifically, patients requiring four or fewer casts achieved significantly better outcomes at both time points, and those with lower baseline Pirani scores experienced more favourable immediate results. These findings align with our study objective by providing robust evidence in support of the Ponseti method as an effective intervention for improving foot deformity in this patient population.

## Discussion

The present study demonstrates that the Ponseti method yields significant improvement in the clinical management of idiopathic congenital clubfoot among neonates, with a marked reduction in the Pirani score from a baseline mean of 3.77 ± 0.73 to 1.61 ± 1.38 immediately post-treatment and further to 0.91 ± 0.81 at the four‐month follow-up. Correspondingly, the proportion of excellent outcomes increased from 47.25% immediately after treatment to 69.23% after four months. These findings corroborate the efficacy of the Ponseti method reported in recent literature over the last decade, which has consistently demonstrated high initial correction rates and sustained improvement with conservative management of clubfoot [15]. Nowadays the Ponseti method is a gold standard that is based on weekly plaster change [10].

The study conducted by Solanki et al. reported excellent and good outcomes in neonates treated with the Ponseti method for idiopathic congenital clubfoot was 75% and 25% respectively [10]. Another study by Mageshwaran et al. reported the successful outcomes after Ponseti management was 85% [16]. Another study reported excellent and good functional outcomes after the Ponseti method were 55.55% and 44.45% respectively [17].

A stratified analysis in our study revealed that clinical parameters such as baseline severity (Pirani score) and the number of casts applied significantly influence outcomes. In particular, patients requiring four or fewer casts had markedly superior outcomes compared to those needing more than four casts (p < 0.001 at both time points). This observation aligns with several contemporary reports that emphasize the importance of early and effective manipulation and casting protocols in achieving optimal correction [18]. It is plausible that a lower number of casts reflects less severe deformities and a more favourable response to treatment, which in turn minimizes the risk of soft-tissue contracture and reduces the need for additional surgical intervention.

This study did not detect a significant influence of demographic factors such as gender, weight, or residence status on treatment outcomes. This finding is consistent with recent studies suggesting that the intrinsic severity of the deformity and adherence to the manipulation and casting protocol are more critical determinants of success than patient demographics [19]. However, age stratification did reveal notable differences, although immediate outcomes between children aged two and younger and those more than two years were comparable (p = 0.110), the older group exhibited a significantly higher proportion of excellent outcomes at the four-month follow-up (88.0% vs. 62.1%; p < 0.001). This somewhat counterintuitive finding may be attributable to factors such as increased tissue responsiveness, greater tolerance of manipulation, or even differences in parental adherence to the bracing protocol among older children. Alternatively, it may reflect a selection bias inherent in our sample, as older children who present later might inherently possess less severe deformities or better tissue elasticity.

When comparing the results with current literature, it is noteworthy that several studies from the past decade have reported success rates with the Ponseti method ranging from 85% to 100% for excellent outcomes, with relapse rates varying between 5% and 20% depending on follow-up duration and brace compliance [20,21]. Our findings of sustained improvement and a substantial increase in excellent outcomes over a four-month period are in keeping with these reports. Nonetheless, differences in reported outcomes across studies may arise from several factors. Variations in the precise manipulation technique, differences in casting materials and protocols, the timing and necessity of percutaneous Achilles tenotomy, and disparities in post-correction bracing protocols can all contribute to heterogeneity in results. Furthermore, socioeconomic factors and levels of parental education may influence long-term brace compliance, which is a well-documented predictor of relapse and overall treatment success [9].

Presently, the Ponseti method is universally recognized as the optimal approach for treating idiopathic congenital clubfoot. Its efficacy, however, is contingent upon strict adherence to the established protocol by skilled practitioners and surgeons [22]. The commitment of parents to the full course of the bracing program is equally critical. Effective and positive communication between the paediatrician and family significantly bolsters brace compliance and, consequently, treatment success [23]. Ponseti programs have now been implemented in many countries with limited resources, bringing us closer to the vision that every child born with clubfoot will have access to high-quality, evidence-based care [24].

The limitations of our study must be acknowledged. The sample size of 91 neonates is relatively small and may limit the generalizability of the results. The non-probability consecutive sampling technique could introduce selection bias [14]. Futhermore, the four-month follow-up period limits us to evaluate long-term outcomes such as relapse rates and functional status. Moreover, our study did not assess factors such as brace compliance or control for potential confounders like socioeconomic status or parental education.

## Conclusions

The Ponseti method is an effective and reliable treatment for idiopathic congenital clubfoot at a tertiary care hospital. Our findings indicate that early and methodical manipulation and casting result in a progressive normalization of the foot, thereby enhancing functional outcomes and promoting a flexible, plantigrade, and pain-free foot. The success of treatment appears to be largely determined by clinical factors, such as the initial severity of the deformity and the number of casts required, while demographic variables seem to exert minimal influence. Future research with extended follow-up periods and broader sample sizes is needed to further evaluate long-term functional outcomes.
